# Insights Into the Older Adults' World: Concepts of Aging, Care, and Using Assistive Technology in Late Adulthood

**DOI:** 10.3389/fpubh.2021.653931

**Published:** 2021-07-02

**Authors:** Wiktoria Wilkowska, Julia Offermann-van Heek, Thea Laurentius, L. Cornelius Bollheimer, Martina Ziefle

**Affiliations:** ^1^Department of Communication Science & Human-Computer Interaction Center, Rheinisch-Westfsche Technische Hochschule Aachen: RWTH Aachen University, Aachen, Germany; ^2^Department of Geriatrics & Department of Geriatric Medicine, Rheinisch-Westfsche Technische Hochschule Aachen: RWTH Aachen University, Aachen, Germany

**Keywords:** aging, assistive technology, technology acceptance, geriatrics, nursing care

## Abstract

The ongoing demographic change forces different stakeholders to cope with increasing needs in nursing care and the economic costs. Consequences arising from the population aging can be supported by assistive technologies to maintain older individuals' autonomy. However, older adults' opinions on the assistance of health-related technologies and their attitudes toward aging and care largely remain underexplored. This paper provides a geriatric and socio-technical perspective, investigating individual perceptions of (a) aging, (b) nursing care, and (c) the adoption of assistive technologies in a cross-national subject group. For this purpose, *N* = 384 individuals (60+ years) participated in an online survey. Findings indicate that most older adults are open to assistive technologies and that individual care preferences contribute to a successful adoption of these technologies. Among individual factors, health status, and gender affect respondents' opinions the most. Our findings help to understand older adults' acceptance of assistive technologies and contribute to the research on the nursing care in private and professional environments.

## Introduction

Increasing life expectancy and the consequences of demographic change have led to considerable challenges in nearly all areas of medicine, with the proportion of individuals in their late adulthood (i.e., aged 60 years and older) steadily growing. This group of “older adults” often requires medical care from several specialized fields of medicine at the same time. Accordingly, what geriatric medicine aims at is the prioritization and integration of measures regarding multimorbidity within an individual care plan. As the increased demand for care is becoming increasingly difficult to meet, this development poses considerable economic and logistical challenges not only from the perspective of medical care, but also in the immediate social environment of the individuals. Older adults are rarely willing to spend the last years of their lives in nursing homes, and thus their families and social environment are often significantly involved.

Modern medical technology—ranging from surgical tools, diagnostic devices, and drug-delivery devices up to complex health-supporting systems in the residential environments of older adults (e.g., ambient assisted living, smart homes)—has revolutionized all areas of medicine (1). When such health-enabling technologies are applied, substantial progress can be made toward more patient-centered care (2–4). Considering the increasing prevalence of chronic disease(s) and complex medical conditions as well as the growing demand for health care delivery resulting from the population aging (5), especially the older part of society can considerably benefit from health-related assistive technologies in their daily lives. The term “assistive technology” refers to ambient and mobile systems and services related to the delivery of assistive products and services, which maintain or improve an individual's functioning and independence, thereby promoting their health and well-being (6). Utilizing such technology may thus prove beneficial not only to the older adults themselves, but also to their caregivers.

Despite the availability of advanced technical solutions, their acceptance and the active use of advanced technical solutions are essential preconditions for meeting the care needs of older adults. Expanding technological capabilities and considering the demographically driven demand are intrinsically connected to the attitudes and perceptions of the potential older users. Among the factors that should be integrated into the decision to voluntarily use assistive technologies are attitudes toward aging and openness to accept health-related assistive technologies (7, 8). Other factors refer to the way societies treat their older adults (9), the economic considerations in the health care supply (5), the cultural and societal norms of aging care in different countries (10), as well as the viewpoints of the older society members themselves. While the variety and availability of assistive devices daily supporting older adults have continuously grown (11–13), older adults' opinions on assistive technologies and attitudes toward aging and care remain underexplored. This article addresses two perspectives: (1) the geriatric perspective that considers elderly individuals and their medical care demands, and (2) the social science perspective that explores the attitudes and perceptions of adults aged 60 years and older regarding (a) aging, (b) nursing care, as well as (c) the acceptance and adoption of health-related assistive technologies in general and related to specific applications.

## Related Work

### Aging—The Geriatric and the Social Perspective

The primary objective of geriatrics is to strive for patient's optimal functionality and autonomy. These goals essentially follow the International Classification of Functioning (ICF) of the World Health Organization (14) with its function-oriented salutogenic and biopsychosocial perspective of health and disease. In an understanding of a “supraspeciality” (15), geriatrics is focusing on the complex interrelationship of concurrent impairments in body function or structure due to age-related multimorbidity with an overall view to the ICF-defined terms of activity (i.e., execution of a task or action) and participation [i.e., involvement in a life situation; (14)]. Due to multiple factors, the key concept of geriatric syndromes are instability, immobility, intellectual impairment, or incontinence (16–18). A proper geriatric treatment is therefore derived from these geriatric syndromes after the underlying factors have been identified and weighed against the preserved biopsychosocial resources according to Baltes' model of Selective Optimization with Compensation in terms of activity and participation (19, 20). Such geriatric treatment goals do not solely rest on pharmacological or surgical measures, but depend on appropriate deployment of assistive devices and nursing support and subsidiary relief. The latter particularly concerns circumstances in which a person is unable to manage their activities of daily living which basically refers to eating, bathing, getting dressed, toileting, transferring, and continence (21). The transition from more or less self-determined material assistance by technical aids and/or medication to an other-directed reliance on nursing care appears decisive in scope.

From the social perspective, aging is a very complex, dynamic, and individually varying process that is connected with losses and gains (22). Negative aspects, such as decline of physical function, dwindling cognitive and mental skills, as well as social isolation often accompany the “elderhood” (23). On the other hand, the today's aging is also associated with higher optimism, higher interpersonal trust, and well-being (24) combined with the growing interest in active and healthy living (25). Thus, the concepts of aging differ individually and the perceptions of a healthy or successful aging must be envisioned as a multidimensional construct (26).

Investigating the older adults' acceptance of technology, there are many reasons to involve subjective, mentally driven concepts of aging into the research. As reported by Kotter-Grühn and Hess, older adults integrate stereotypical information into their self-perceptions of aging (27). This can have positive consequences, as positive perceptions through the prism of a valuable life experience and life wisdom can lead to a high level of satisfaction and experiencing aging as very fulfilling. But integrating stereotypical information can be also problematic, because the activation of self-relevant age stereotypes can influence the older adults' performance. Another study revealed that old people (90+ years) emphasize the importance of having one's own home and living independently there as long as possible; they also prefer to have a quick and easy death rather than being institutionalized (28). However, living independently can be quite challenging for older adults, especially when lived with functional impairment(s) and/or chronic disease(s) reflected in the notion of a geriatric phenotype and frailty (29). A chronic health condition often results in the need of care support from the family or professional healthcare services and the higher demand for medical interventions and care for older adults causes the necessity to improve the amount and quality of gerontological nursing (30). In the following, we understand nursing care as the support and care for older adults in need of care in both private/family settings and professional care contexts (e.g., hospitals, nursing homes), depending on (i) the severity of their condition and therefore the therapeutic support required, (ii) the resulting need for support, and (iii) the accessible care options.

For older adults, the perceptions of their health condition may be not only limited to the acute illness experiences, but may also include the understanding of a changing state of health and well-being that is managed and supported through the use of multiple, assistive technologies, and environmental design modifications (31). Correspondingly, to avoid the expensive institutional nursing care of older adults and to provide means to cope with the anticipated shortage of care professionals, technology can effectively assist (32). Today, older adults are increasingly more technology-prone in digital settings and capable of utilizing technological artifacts for their own needs (31). In order to better understand the mechanisms behind the technology adoption by autonomously living older adults, it is crucial for research to consider their attitudes toward aging and individual preferences regarding care measures.

### Health-Supporting Technologies and Users' Acceptance

Technical development in health-supporting technologies—including innovations in the areas of ambient assisted living, lifelogging technologies, and gerontechnology (henceforth also referred to as “assistive technologies”)—have generated an increasing number of devices and systems in recent years, being able to support older individuals and people in need of care (11–13). In more detail, it is possible to support these people in their autonomy, having an active lifestyle, and staying within the own home as long as possible, whereas at the same time also the (in)formal caregivers can be relieved (33–35). To name a few examples, health-supporting assistive technologies can be realized as smart home systems (36), as (wearable) sensors (37), or as video-based systems (13), and can fulfill in particular medical safety-relevant functions, e.g., detection of emergencies or monitoring of vital parameters (11). Even beyond that, the monitoring of a person's vital parameters enables to detect changes in the health status, behavior, movements/gait, or sleep rhythm, which can indicate (age-related) diseases, such as dementia or Parkinson [e.g., (38)]. Other functional areas aim at providing support in cognition and memory (39) or facilitating social interaction and communication with family members or medical staff (40).

According to the technical development, empirical research has analyzed the acceptance and adoption of these technologies by a prediction of technology usage, especially applying the Technology Acceptance Model and the Unified Theory of Acceptance and Use of Technology (41, 42). These models were adapted, extended, and applied for diverse contexts, health-related applications, and user groups (43).

Research in the field of health-supporting technology acceptance predominantly revealed approving attitudes among the users [e.g., (44, 45)], identifying relevant usage motives and barriers [e.g., (44, 46, 47)]. In addition to the influencing factors identified in the theoretical models, several studies focused on the acceptance of health-supporting technologies for specific user groups [e.g., (22, 43)] and the impact of the users' individual characteristics on acceptance: People's age can significantly impact their attitudes toward aging [e.g., (22)], which in turn influences the willingness to use health-supporting technologies (48). Also gender was found to significantly impact technology acceptance in the health-related context [e.g., (3, 49)]. Not least, people's health status as well as potential health impairments were identified as influencing factors for the perceptions of aging and the acceptance of health-supporting technology [e.g., (50, 51)].

### Questions Addressed

Beyond the mentioned relationships, the empirical research has barely considered the combination of factors referring to the older individuals' perceptions of (a) aging, (b) nursing care as well as (c) their acceptance of using assistive technologies so far. To address this gap, present research intends, on the one hand, to demonstrate the gerontological point of view on older adults' age-related functional impairments and their underlying diseases, and, on the other hand, to empirically explore the social perspective in this age group. For this purpose, older males and females with different health conditions were surveyed regarding relationships between their individual characteristics and perceptions of aging, nursing care, and the use of health-supporting technology. The research questions address the following aspects:

- What are older adults' attitudes toward aging? (RQ1)
- What are older adults' preferences and their readiness for nursing care? (RQ2)
- How do older adults accept health-related assistive technology in general? (RQ3)
- To what extent are specific technological applications accepted among older adults? (RQ4)

To realize these objectives, an international target group was surveyed. This methodology provides not only a higher diversity among the older adults and therefore more valid empirical statements, but it also enables to gather opinions reaching beyond the local and cultural conditions of only one country.

## Methods

### Measures and Research Design

**Figure 1:**
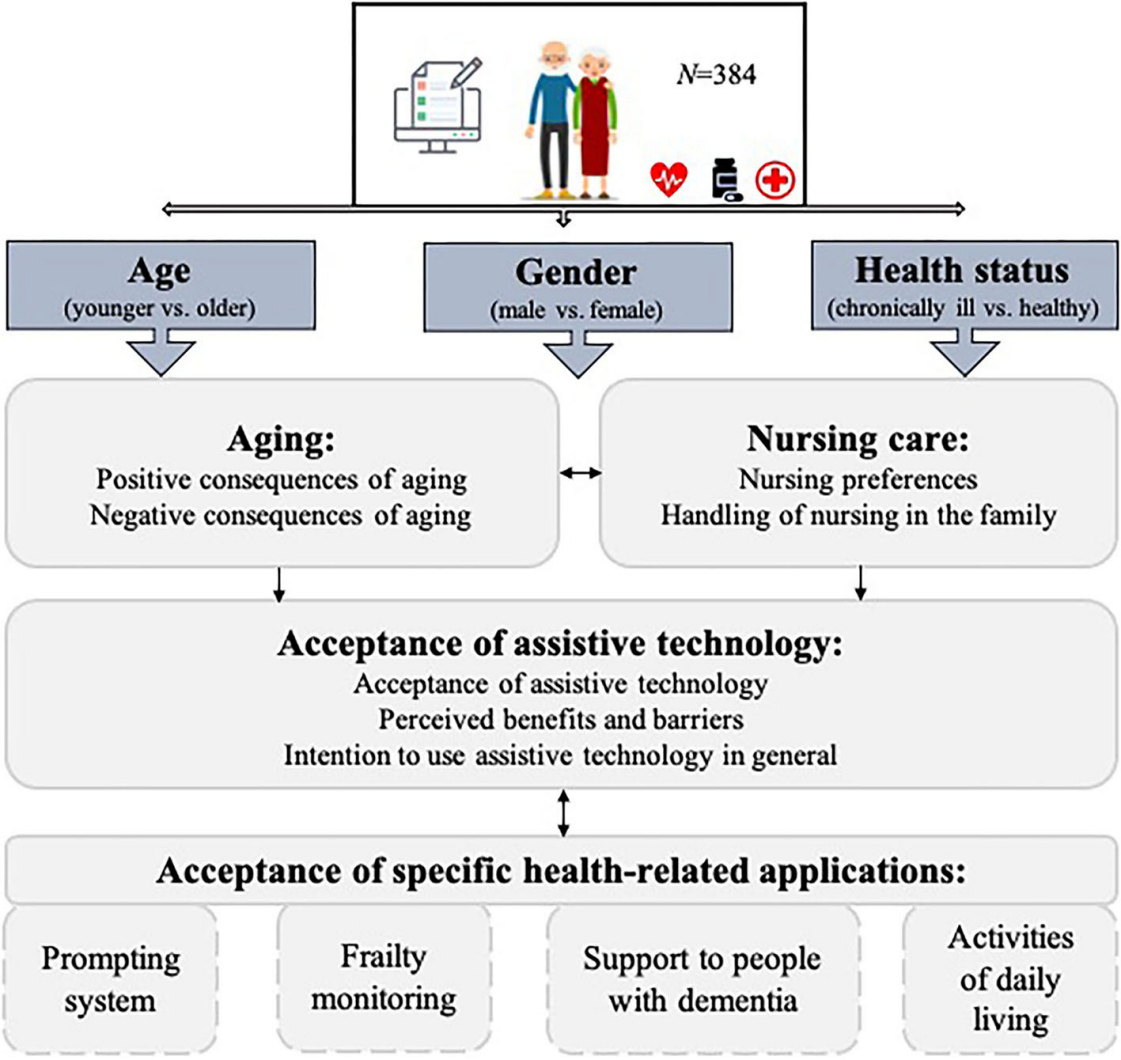
Research design of the study.

The constructs used for the investigation of the questions addressed are summarized in Figure 1.

This study used a quantitative research method in the form of an online survey. One of the dependent variables represented the assessments of positive and negative consequences of aging, such as health vulnerabilities, less social contacts, but also more experience in life and the attitudes resulting from the valuation of these consequences. In addition, the survey examined general preferences and the handling of nursing care as well as the acceptance of assistive technologies. As regards the latter, the technology acceptance in a general sense and specific assistive applications were taken into account.

As independent variables, the study examined the impact of three individual user characteristics: The first factor refers to the participants' age [younger (60–69 years) vs. older seniors (70+ years)], since it could be argued that people's attitudes change fundamentally with the increasing age due to the growing number of complaints or deficiencies induced by aging itself. The second influencing factor is the participants' health status (healthy vs. chronically ill), which has been proven to affect individuals' opinions in health-related matters. And the third factor is the participants' gender (female vs. male), as it is not only to assume that women and men have different approaches to aging, but it is also widely known that both gender groups deal with technology in different ways.

A European research project PAAL (“Privacy Aware and Acceptable Lifelogging services for older and frail people”) was the initiator for the present study: An interdisciplinary team of lawyers, psychologists, engineers, computer, and communication scientists from Sweden, Germany, Spain, Italy, and Canada have developed assisting lifelogging applications that are specifically tailored to the needs and requirements of older users. To gain representativeness among the participants over different European and North American countries, the service of an independent international market research institute was used to collect the data from the online survey. For recruitment purposes, appropriate quotas were required with regard to the participants' age and country of origin. Participants were paid for their participation by the survey panel's institute and it took them 15 min on average to complete the survey. The participation was voluntary and the participants could not proceed with the online survey unless they indicated their consent to participate at the start of the survey.

### Structure of the Online Survey

Based on previous research and partly validating the findings [i.e., (22)], we constructed an online survey which focused on perceptions of aging and care as well as acceptance of health-assisting technologies in one's old age. To reach an international audience, the online survey was performed in four languages including German, English, Spanish, and Italian.

The survey consisted of three parts: In the first part, participants indicated their demographic information, such as their country of origin, age, gender, educational level, income, and housing situation. Using the “Subjective Vitality Scale” (52) respondents assessed their feelings of energy and vitality (e.g., “I look forward to each new day”; “I feel alive and vital”) on a 6-point Likert-type scale (1 = “not at all true” to 6 = “very true”). They furthermore answered questions regarding their health condition (e.g., “I am perfectly healthy,” “I suffer from a chronic illness, but I can cope with it well”), indicated if they suffer from a chronic illness and if they need a caring assistance in their everyday life (“yes”/”no”). The following questions referred to the professional and private experience with care (answer options: “yes”/”no”) and to the participants' attitude toward caring for frail family members (e.g., “In my family, older family members, and/or those in need of care are cared for by the family at home”; 1 = “I disagree” to 4 = “I agree”).

The second part of the survey started with self-developed questions regarding perceptions on the positive (5 items; Cronbach's α = 0.83) and negative consequences of aging (5 items; Cronbach's α = 0.80), for example, “Getting older means to me that… because of my life experience I can still be very useful to the society and my family”; “…I feel lonely more often.” The whole scale regarding attitude toward aging consisted of 10 items and showed satisfactory internal consistency of α = 0.82. After that, participants evaluated statements referring to their preferences in the situation when they would rely on support and care (e.g., “I would be glad to have a professional care service supporting me”). The scale of nursing preferences was composed of 8 items (Cronbach's α = 0.83). In the next step, a short scenario introduced the situation that using health-related assistive technology in case of a chronic illness could take over some part of, or partly supplement, the work of the (in)formal caregivers. The participants were asked to empathize with the situation that they are in need of care themselves and that an ambient lifelogging technology has the potential to assist and facilitate their everyday life in their domestic environment by detecting emergencies such as falls or by reminding for daily routines (e.g., intake of medicine or appointments). Here, participants evaluated the perceived advantages (e.g., fast reaction in case of emergency, increased autonomy) and disadvantages (e.g., dependency on the system, isolation). The overall scales consisted of 9 items each, generating very high internal consistencies (pros: α = 0.95; cons: α = 0.90). Three further questions referred to the general intention to use assistive technology for health-related purposes (e.g., “I can well-imagine the use of a health-supporting home assistance system”) and formed a consistent scale (α = 0.75). For all items in the second part of the survey, the answer options ranged from 1 (“I fully disagree”) to 6 (“I fully agree”).

The third part of the survey evaluated the participants' willingness to use specific assistive applications and functions (“I would like to use the following functions and corresponding technologies”): (i) prompting system (i.e., video-camera on the ceiling to alert care staff in the event of untypical behavior or events, like longer absence), (ii) frailty monitoring (i.e., health analysis for older people using a networked gripping ball for measuring muscle strength and its progression), (iii) support to people with dementia (i.e., sensors integrated in the bed for alerting care stuff in case of untypical behavior, such as not getting up), and (iv) recognition of activities of daily living (i.e., room cameras for recognition and analysis of daily activities).

### Description of the Participants

The age of the participants ranged between 60 and 99 years (*M* = 67.6, *SD* = 5.5) and 52% of the sample were female. The international sample included participants from a Northern European country (Sweden: *n* = 92; 24%), a Central European country (Germany: *n* = 85; 22.1%), Southern European countries (Spain: *n* = 51; 13.3%; and Italy: *n* = 49; 12.8%), and a North American country (Canada: *n* = 107; 27.9%), which were chosen rather arbitrarily, guided by the country affiliation of the project partners in the aforementioned framework project PAAL. Most of the respondents (*n* = 198; 51.6%) reported to have completed the middle level of education (high school, apprenticeship), followed by the holders of a university degree (*n* = 151; 39.3%) and the smallest part (*n* = 49; 12.8%) indicated to accomplish the low level of education (*n* = 35; 9.1%).

Regarding the general state of health, more than half of the sample (52%) stated to be in a very good health condition (*n* = 200). A large part of the sample (46.6%) also reported to suffer from at least one chronic illness: 32.3% of them did fine on their own (*n* = 124) and the other 14.3% indicated to be partly restricted (*n* = 55). The mean level of the self-reported vitality resulted by *M* = 29.6 (*SD* = 5.5; *min* = 8, *max* = 42). This result suggests that the majority of the participants tended to experience positive feelings of aliveness, enthusiasm, and vigor (53)—perceptions being functionally significant for human motivation and well-being in the everyday life. Additionally, we found a strong effect of the health status (see Table 1) in this regard: Healthy individuals reported decisively higher levels of vitality in comparison to the chronically ill respondents.

**Table 1:** Statistical results for the *significant* effects of the user factors on the perceptions of aging, nursing care, and acceptance of assistive technologies (*N* = 384); *CA* = Cronbach's alpha.

			**User factors [M (*****SD*****)]**							
			**Senior groups**		**Gender**		**Health condition**			
	**Construct**	**Item examples**	**Younger** **(60–69)**	**Older (70+)**	**Female**	**Male**	**Healthy**	**Chronic**	**Statistics of differences**	**Effect size**
Age and care	Subjective vitality scale (7 items; *CA* = 0.92; *max* = 42)	“*Sometimes I feel so alive, I just want to burst*”	–	–	–	–	32.6 (5.6)	26.3 (6.8)	*F*_(1, 384)_ = 82*, p* ≤ 0.001	η^2^ = 0.18
	Attitude toward aging (10 items; *CA* = 0.82; *max* = 60)	“*Getting older means to me that I become more relaxed about a lot of things*”	–	–	–	–	46.7 (6.8)	41.6 (6.9)	*F*_(1, 384)_ = 44.8*, p* ≤ 0.001	η^2^ = 0.11
	Positive consequences of aging (5 items; *CA* = 0.83; *max* = 30)	“*Getting older means to me that I can cope better with adversity through my experience*”	–	–	–	–	24.3 (3.5)	22.1 (4.1)	*F*_(1, 384)_ = 25.9*, p* ≤ 0.001	η^2^ = 0.06
	Negative consequences of aging (5 items; *CA* = 0.80; *max* = 30)	“*To me, getting older means less independence*”	–	–	–	–	12.6 (4.7)	15.6 (4.5)	*F*_(1, 384)_ = 33.9*, p* ≤ 0.001	η^2^ = 0.08
	Preferences for nursing (8 items; *CA* = 0.83; *max* = 48)	“*I would like to be able to do things more independently*”	–	–	36.4 (6.2)	34.4 (6.6)	–	–	*F*_(1, 384)_ = 9.8*, p* = 0.003	η^2^ = 0.02
	Handling of care in the family (max = 6)	“*In my family it goes without saying that (older) family members are cared for by the family at home*”	2.7 (1.1)	2.4 (1.1)	–	–	–	–	*F*_(4, 373)_ = 2.5*, p* = 0.040	η^2^ = 0.03
Acceptance of assistive technology	Perceived pros (9 items; *CA* = 0.95; *max* = 54)	“*Using assistive technology is a gain to safety*”	–	–	–	–	41.4 (7.6)	39.6 (8.0)	*F*_(1, 383)_ = 4.8, *p* = 0.029	η^2^ = 0.01
	Perceived cons (9 items; *CA* = 0.90; *max* = 54)	“*The technology stigmatizes its users by visualizing their illnesses*”	–	–	34.1 (9.1)	31.6 (7.9)	–	–	*F*_(1, 383)_ = 8.5, *p* = 0.003	η^2^ = 0.02
	Acceptance of using assistive technologies (3 items; *CA* = 0.75; *max* = 18)	“*In general, the usage of home assistance systems is useful*”	–	–	–	–	–	–	n.s.	–
	Specific assistive applications and functions	*Prompting system* (4 items; max = 24)	–	–	–	–	15.6 (4.4)	14.1 (4.4)	*F*_(1, 383)_ = 12.3, *p* ≤ 0.001	η^2^ = 0.03
		*Frailty monitoring* (4 items; max = 24*)*	–	–	–	–	16.4 (4.2)	14.8 (4.2)	*F*_(1, 383)_ = 14.2, *p* <0.001	η^2^ = 0.03
		*Support to people with dementia* (2 items; max = 12)	–	–	–	–	8.2 (2.2)	7.3 (2.4)	*F*_(1, 383)_ = 16.1, *p* <0.001	η^2^ = 0.04
		*Recognition of the activities of daily living* (3 items; max = 18)	–	–	–	–	10.8 (3.5)	9.9 (3.3)	*F*_(1, 383)_ = 6.3, *p* = 0.013	η^2^ = 0.02

Overall, 10% of the participants (*n* = 38) confirmed to rely on the support and care from relatives and professionals. Regarding experience with care, 17.4% (*n* = 67) of the respondents indicated to have a professional experience in the field of nursing. Private experience with nursing reported *n* = 96 of the survey participants (25%), who referred to passively witness person(s) in need of care within their family circle, and 37.5% (*n* = 144) reported to have been an active caregiver for a family member.

### Data Analysis

After data cleansing, excluding incomplete data sets, speeders, and implausible answer patterns (*n* = 168; 33%), data of *N* = 384 respondents were included in the statistical analyses. We report descriptive statistics using means (*M*) and standard deviations (*SD*). To examine differences regarding the user factors, we applied (multivariate) analyses of variance [(M)ANOVA]. For effect size measures, the parameter partial eta squared (η^2^) is reported according to Cohen (54). The statistical significance (*p*) was set at the conventional level of 5%. All relevant statistical parameters of the performed analyses are summarized in Table 1.

## Results

### Perceptions of Aging and Care

#### Positive and Negative Effects of Aging

**Figure 2:**
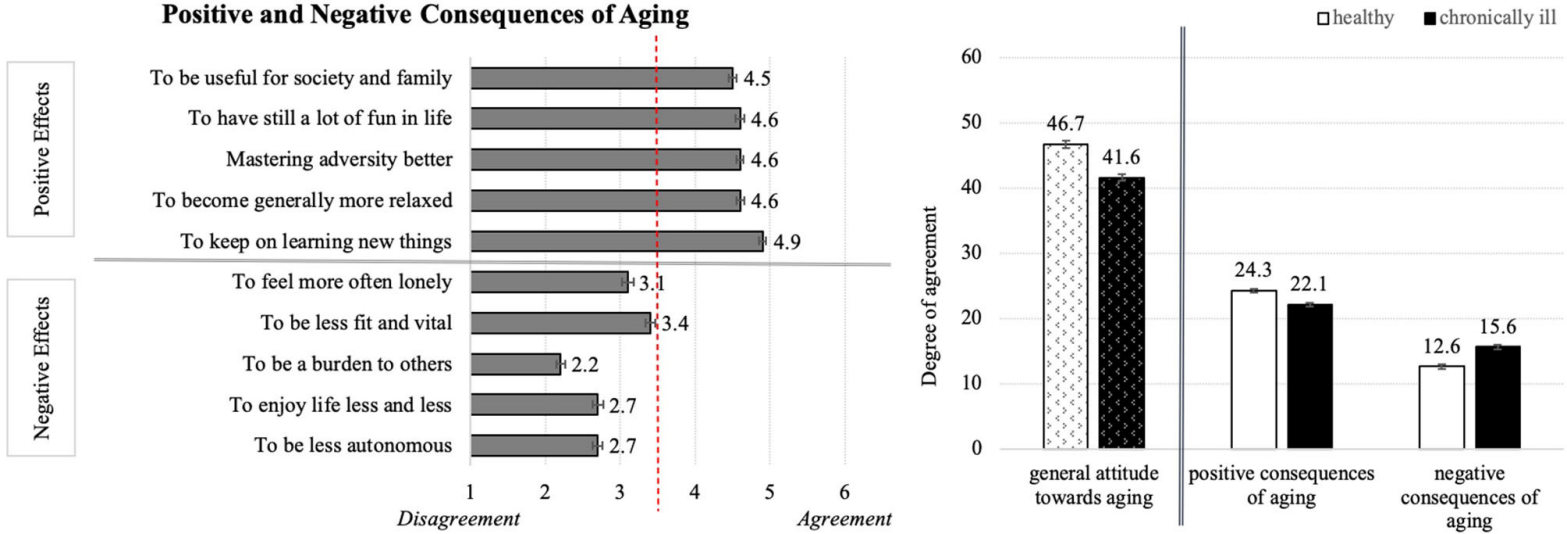
Perceptions of positive and negative consequences of aging **(Left)** and the effect of health status on the general attitude toward aging (**Right**; *N* = 384).

Depicting the means of the entire sample (Figure 2, left), positive consequences of aging reached generally higher values than the negative consequences. These results point to a thoroughly positive attitude toward aging, with participants recognizing the strengths associated with aging and tending to deny the uncertainties and inconveniences. In more detail, all statements referring to the positive consequences of aging were almost equally approved by the participants, whereas the aspect “to keep on learning new things” received the highest agreements. Referring to the negative consequences of aging, the item “to be less fit and vital” reached the highest mean value and “to be a burden to others” reached the lowest value. Yet, perceptions of all negative consequences of aging were on average (slightly) rejected.

Univariate analysis of variance including the factors age, gender, and health status, revealed a significant effect of health status on the perceptions of positive and negative consequences as well as the general attitude toward aging. Healthy individuals reached overall higher attitude toward aging than chronically ill persons (Figure 2, right). This moderate effect (η^2^ = 0.11) is mirrored in the significant differences in the perceptions of positive and negative aspects of aging: Healthy respondents evaluated the positive consequences on average significantly higher and the negative consequences significantly lower than the chronically ill persons. As opposed to this, the factors age and gender did not affect the opinions about aging.

In sum, younger and older participants of both gender groups demonstrated more positive than negative attitude toward aging and the health condition significantly influenced the perceptions of aging.

#### Nursing Preferences

**Figure 3:**
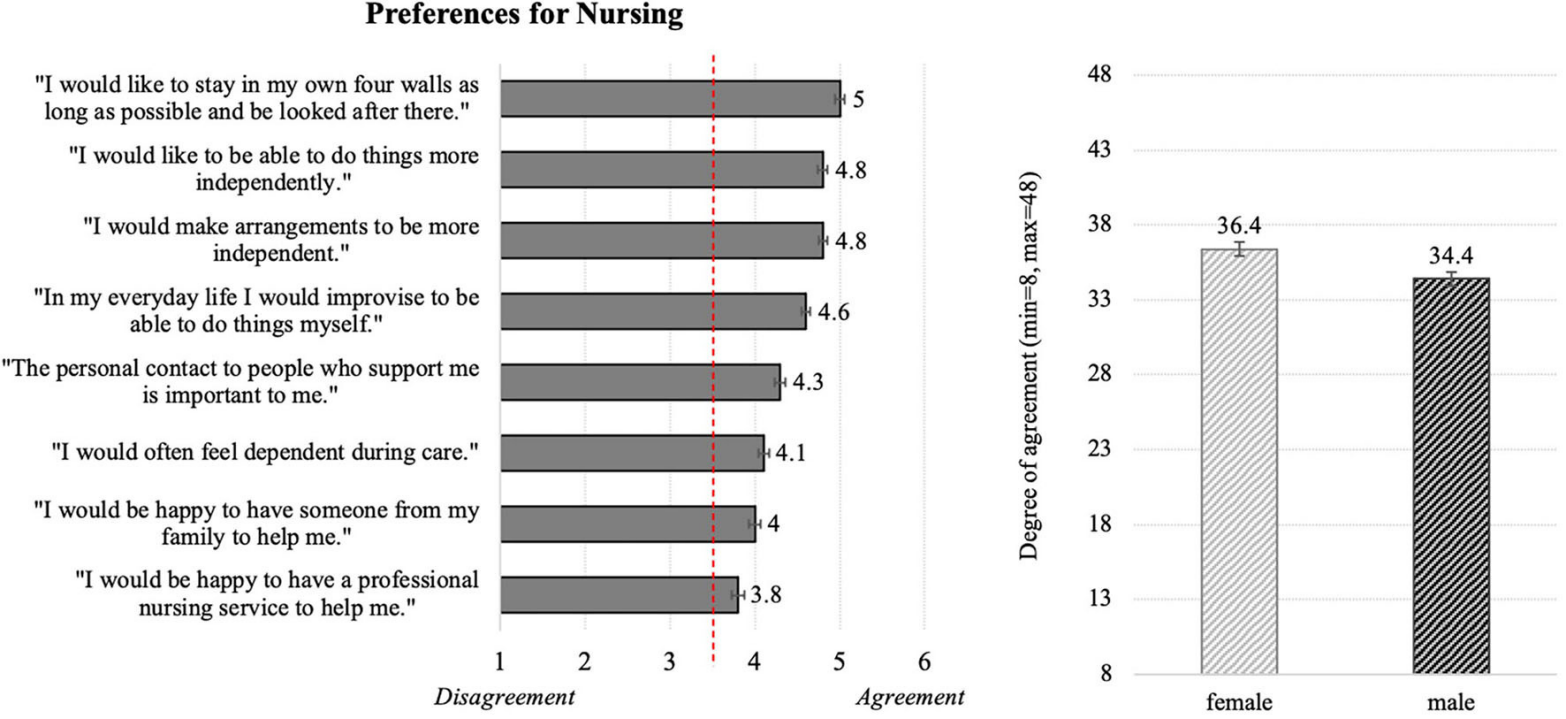
Descriptive statistics for the evaluations of the general nursing preferences **(Left)** and the effect of gender on the nursing preferences (**Right**; *N* = 384).

Figure 3 (left) depicts the resulting means for the preferred conditions of nursing care and indicates that most respondents desire to stay in their own home environment surrounded by their family as immediate caregivers, managing their everyday life as far as possible autonomously. Although people tended to agree to a professional nursing service in case of a disease, they were aware of the intrinsic dependency. Diving into details, all single statements received approval by the participants, while the wish to stay at home as long as possible and statements regarding independency reached on average the highest agreements. In comparison to that, the concrete desires to receive help from the own family or from care services received clearly lower, but still positive values.

In addition, ANOVA revealed a significant impact of gender on the scale referring to the nursing preferences. According to this result, females agreed more to the below-mentioned statements in comparison to males (Figure 3, right).

#### Handling of Care in the Family

**Figure 4:**
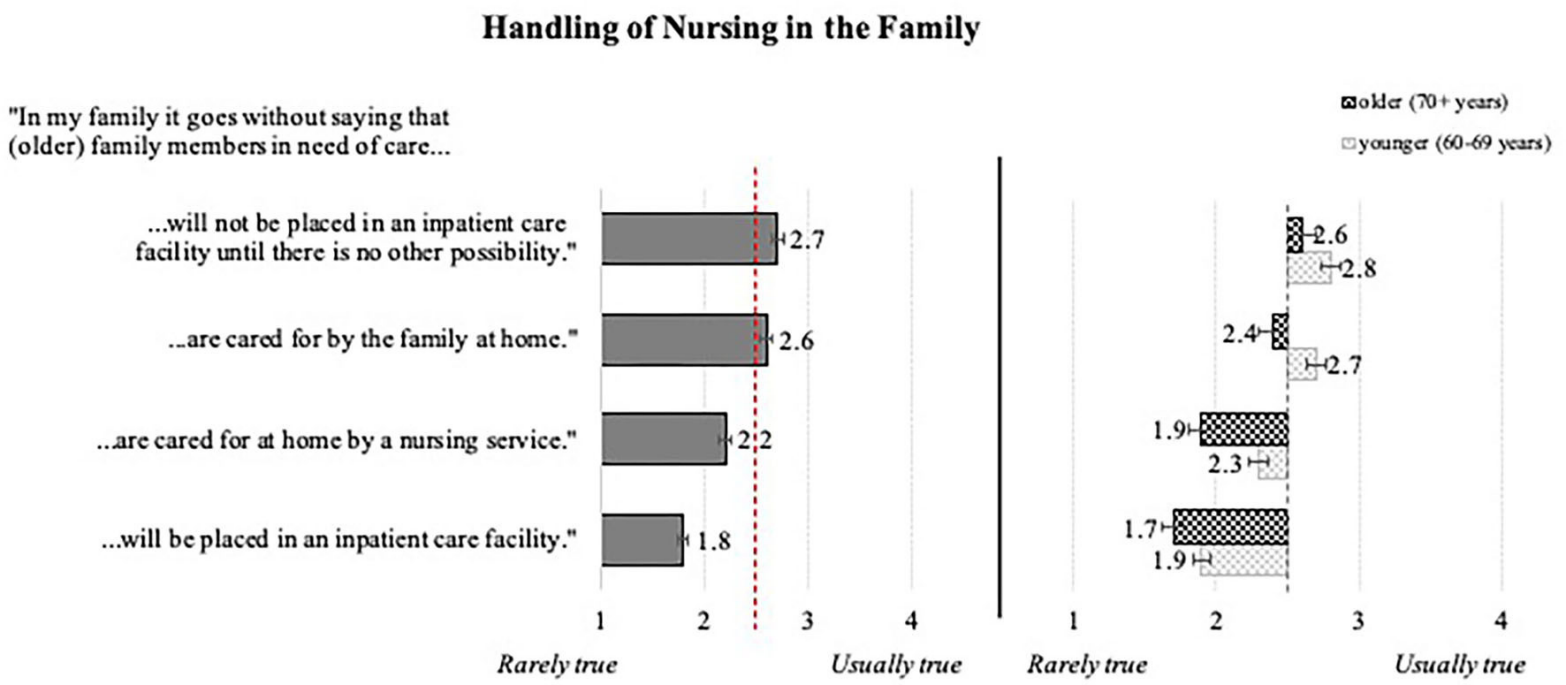
Evaluations of the handling of nursing in the family: overall means **(Left)** and effect of age (**Right**; *N* = 384).

Scores for the handling of care within the immediate family (Figure 4, left) are ambiguous, oscillating around the middle of the answer scale. Among the four statements, the aspect of not placing older family member(s) in need of care in “an inpatient care facility until there is no other possibility” reached the highest but still rather neutral evaluation. In contrast, the statement “will be placed in an inpatient care facility” received the most “rejecting” evaluation.

Examination of significant influences of the personal characteristics (MANOVA) suggests a weak impact of the chronological age in terms of nursing cared out by the family. As is depicted in Figure 4 (right), younger participants reached in all items higher means than the ones aged 70 years and older. Interestingly, the overall health status did not play a significant role in this regard.

Summing up so far, individual factors (age, gender, and health condition) influence attitudes toward aging and care only selectively; this is reflected by only weak to moderate statistical effects.

### Preparedness for Using Assistive Technology

#### General Acceptance

**Figure 5:**
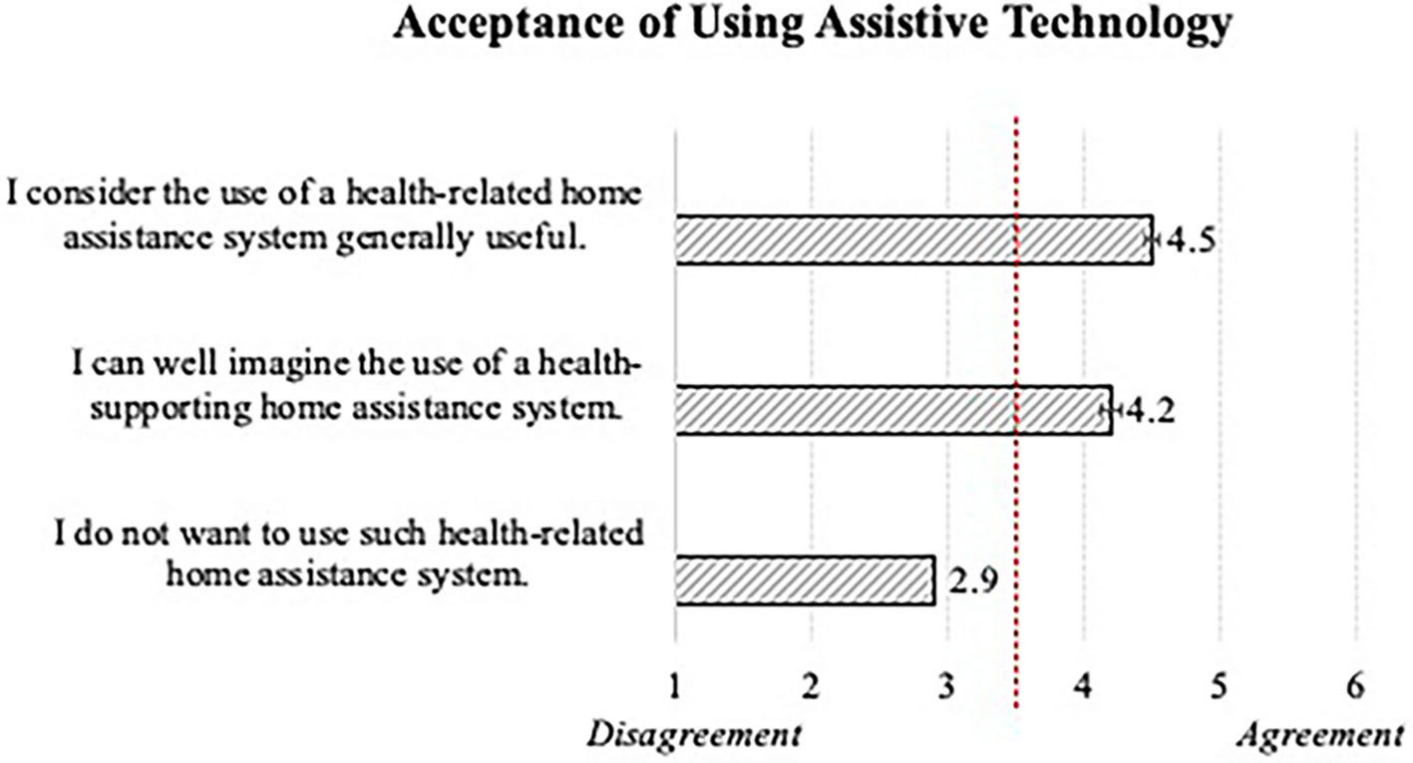
General acceptance of using assistive technology in older adults' everyday lives (*N* = 384).

In terms of the general acceptance of using assistive technology meant to support older adults to manage their daily necessities (e.g., dietary schedule, medication intake, exercising, etc.), participants' assessments revealed a positive attitude and willingness of utilization (Figure 5). On the level of the single statements, participants confirmed the usefulness of the health-related home assistance system and showed a general preparedness to use it. The means resulted for men and women in both age groups and independently from their health condition, which indicates no relevant influences of the individual characteristics on the technology acceptance in the health-supporting context.

**Figure 6:**
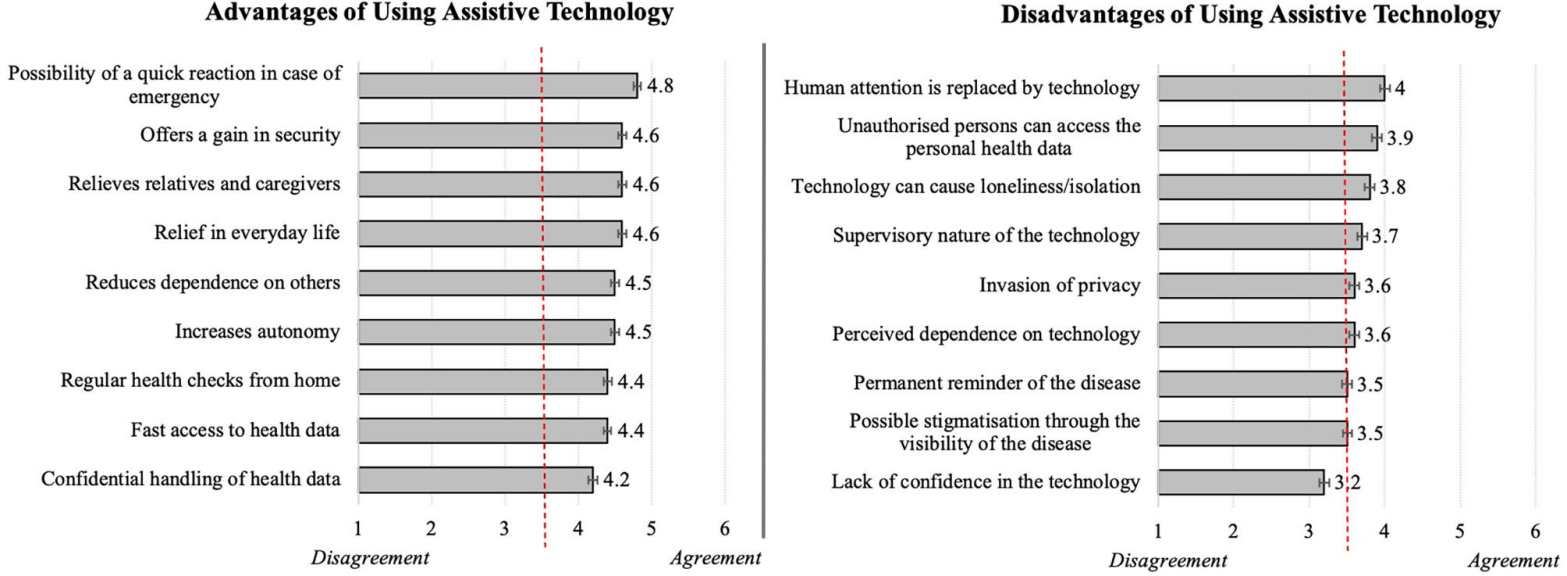
Evaluations of advantages **(Left)** and disadvantages **(Right)** of using assistive technology (*N* = 384).

As depicted in Figure 6, the respondents agreed to all items referring to the perceived advantages (left) of using assistive technology in private environments, attesting a quite positive attitude. The ratings revealed very similar means for the statements surveyed: The “possibility of a quick reaction in case of emergency” was perceived as the biggest advantage and the aspect of the “confidential handling of the health data” received on average the lowest, but still approving evaluations.

As to perceived cons (Figure 6, right), the resulting opinions which oscillated around the middle of the scale, were rather neutral; thus, participants neither clearly confirmed nor clearly denied them. Here, the concern that the “human attention is replaced by technology” reached the highest evaluations representing the most relevant disadvantage, while the “lack of confidence in the technology” was revealed as the least relevant disadvantage of using health-related home assistance systems.

**Figure 7:**
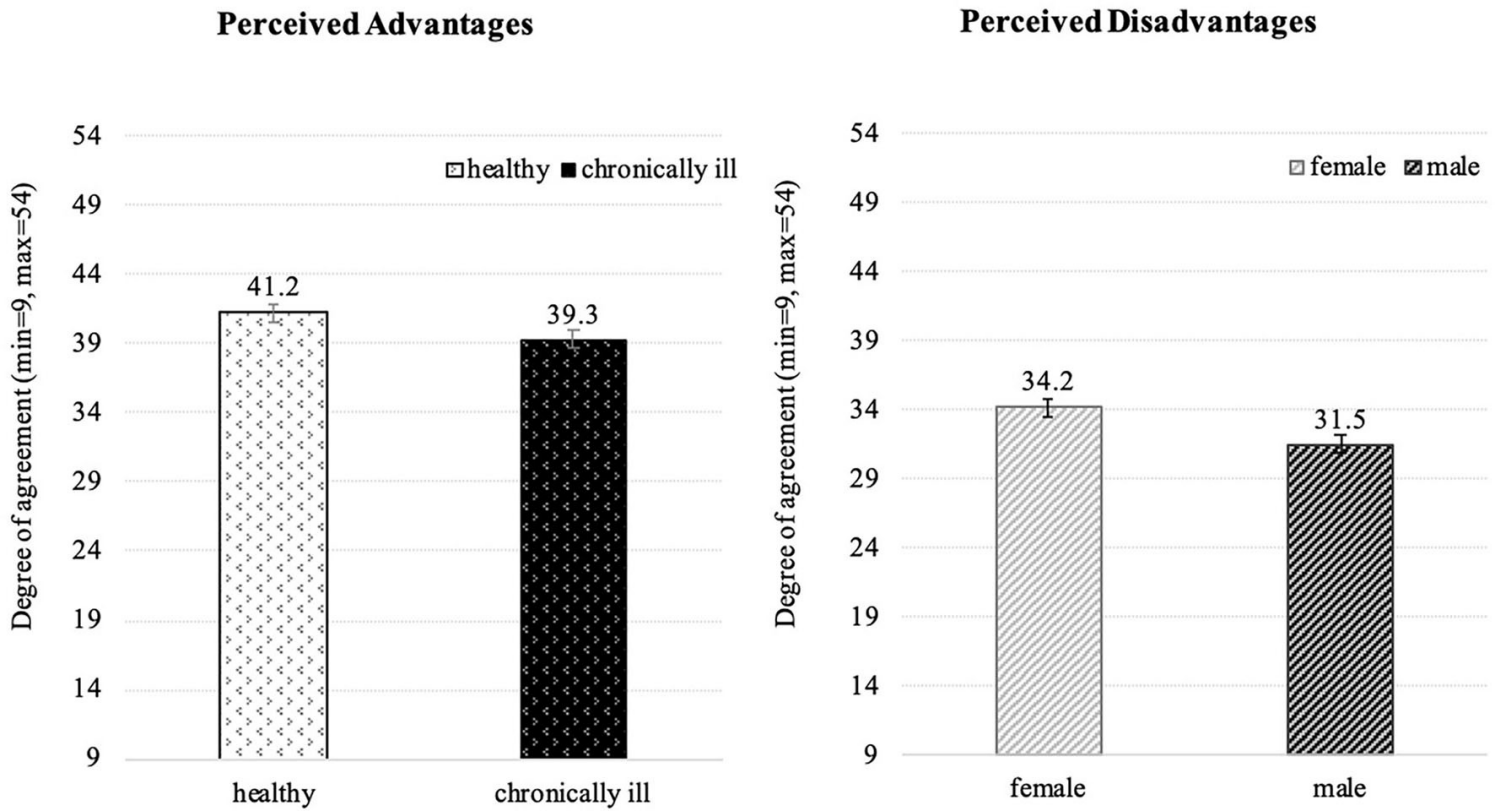
Influence of health status on the perceived advantages **(Left)** and impact of gender on the perceived disadvantages **(Right)** of using assistive technology (*N* = 384).

Moreover, in this context a significant impact of gender and health status was revealed (see Figure 7). According to analyses of variance, healthy persons perceived the use of assistive technologies slightly more positive than chronically ill respondents. Also, women more than men agreed with perceived disadvantages of assistive technology. In contrast, younger vs. older seniors did not significantly differ in their opinions.

#### Acceptance of Specific Assistive Applications and Functions

**Figure 8:**
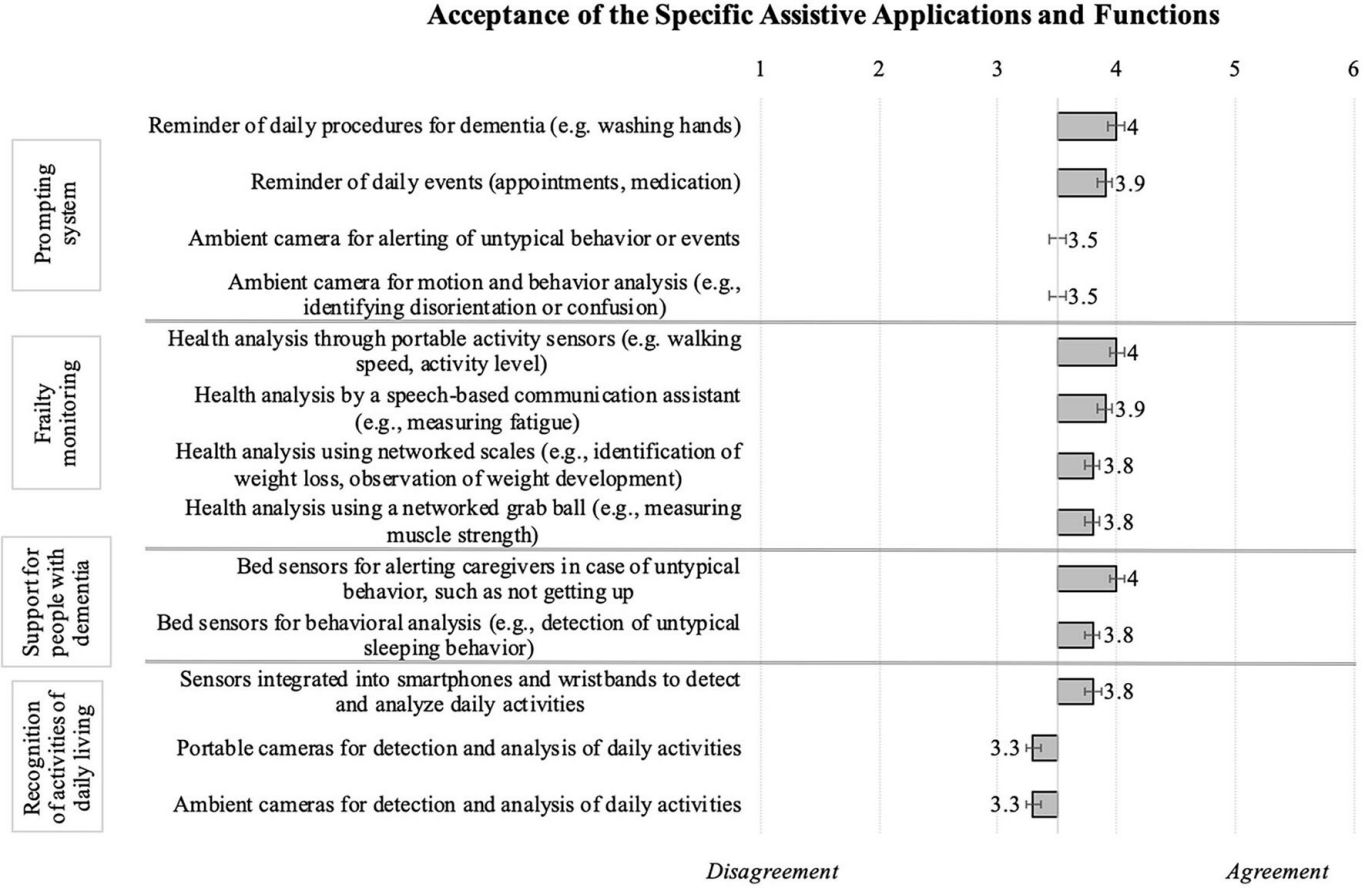
Technology acceptance of specific health-related applications and functions (*N* = 384).

Finally, participants rated specific technological applications considered helpful in the everyday life of older individuals or persons suffering from illnesses particularly occurring in older age, for instance dementia. Figure 8 summarizes all resulting means: The most accepted technical applications were the frailty monitoring and functions supporting people with dementia (note: the participants themselves did not indicated to suffer from dementia!). Whereas reminder functions of a prompting system as well as built-in sensors monitoring daily activities were generally appreciated, cameras were either rejected or, at best, neutrally evaluated. To be more specific, the prompting system that provides reminders for daily procedures or events like washing hands or medication intake, reached a higher acceptance than ambient cameras for detecting/analyzing untypical behavior. In addition, respondents positively evaluated applications regarding frailty monitoring, enabling health analyses (e.g., walking speed, activity level), speech-based communication, observation of weight development, and measurements of muscle strength. Assistive technology supporting dementia patients and their caregivers (e.g., bed sensors for behavioral analyses) was also endorsed. Yet, looking at the evaluations of assistive technology that recognizes activities of daily living, participants readily accepted sensors integrated in smartphones or wristbands, while they rejected using portable and ambient cameras.

**Figure 9:**
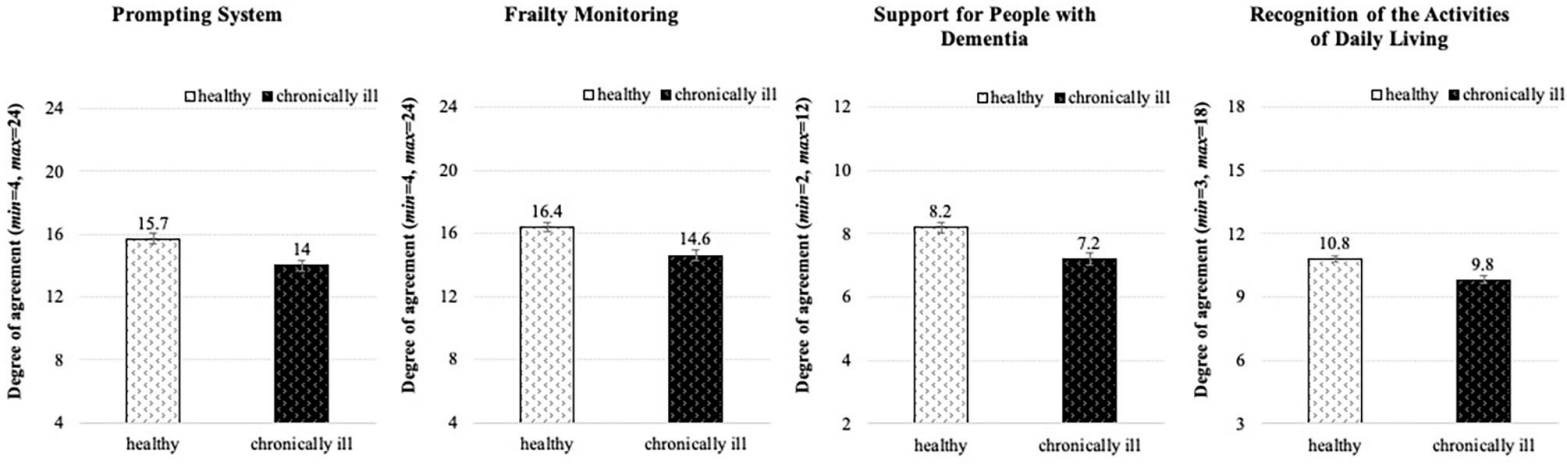
Effect of the health status on acceptance of using specific assistive applications and functions (*N* = 384).

Statistical analysis of the impact of personal variables revealed that especially the individual health status significantly affected the perceptions and anticipated use of the specific applications and functions (see Table 1). This effect is depicted in Figure 9: In all investigated cases healthy participants reached decisively higher agreement on the use of functions and corresponding technologies than chronically ill individuals. Besides, we found no significant differences between the age groups and the gender groups.

In summary, it can be stated that elderly persons generally posed an accepting attitude toward assistive technologies. They basically perceived more advantages than disadvantages of the technology, however, respondents of both genders and depending on their health conditions differed significantly in their opinions. Also, specific applications and functions that enable health monitoring and support the everyday routine encountered acceptance; here, healthy individuals showed higher willingness to use the technology than the chronically ill respondents. In contrast, technology that uses cameras for the daily assistance was mostly rejected.

## Discussion

The aim of this study was to enhance knowledge about the opinions on assistive technologies and nursing support that particularly concern individuals with increasing age and the imminent danger of a geriatric phenotype. We examined the perceived consequences of aging, nursing care preferences, and attitudes toward using health-related assistive technologies. Older adults (60+ years) from five countries (Germany, Spain, Italy, Canada, and Sweden) were included as respondents to our online survey.

### Summary of the Key Results

Findings revealed that individual factors of age, gender, and health condition influence attitudes toward aging and care only selectively. Healthy individuals, as opposed to chronically ill persons, perceive advantageous changes induced by aging significantly more and the rather disadvantageous consequences of aging as significantly less negative. This outcome is not surprising, but it provides an important evidence on how health condition can massively impact the vital aspects of one's own life. Older adults with such positive self-perceptions of aging more likely allow the support of assistive technologies in order to maintain their social connectivity (55) and autonomous living in everyday life as long as possible (56).

Most participants confirm the will to live independently and, if care is necessary, it is desired within the family circle. Personal contact with caregivers is important, preventing social isolation, loneliness, and possible consequences associated with mental health, such as depressions and anxiety (57). According to the results, women?more than men?wish to stay in their “private four walls” as long as possible and emphasize highest possible autonomy in case of the condition that requires a special nursing care. The results are in good alignment with previous research that emphasizes a particularly strong attachment of older women to their homes and possessions (58). However, these gender-specific findings might also originate from traditional family roles, in which women regularly take over the task of parenting and housekeeping, including also the care of vulnerable family members. Furthermore, we found significant age differences in how handling of care within the family is perceived: Individuals aged 70 years and older are less optimistic about nursing within their family in comparison to their younger counterparts. This result may correlate with the significant increase in age-related health issues.

In addition, outcomes referring to technology acceptance confirm a fundamental preparedness to employ them, both in general and in specific applications targeting assistance for older users. Especially healthy individuals appreciate the advantages of such technological assistance increasing their safety, controlling their health problems, and compensating for their functional impairments. Nevertheless, the participants—females more than males—have also reservations about the technology, fearing that human attention replaced by the technology could cause loneliness or social isolation. This result validates previous findings, which showed that technologies can enhance the older adults' quality of life but otherwise also lead to reduced face-to-face communication and social exclusion (59). As further disadvantages, respondents were somewhat apprehensive of the supervisory character of the technology and risk of misuse of their sensitive health data. Nonetheless, participants agree to utilizing such devices when asked about applications supporting persons with age-related illnesses, for example dementia, and they are willing to use specific functions, such as health monitoring and reminder of daily events or duties (e.g., medication). This consent however does not apply to the use of cameras, which were vigorously rejected by the participants of the present study?a result that again corroborates previous findings (60). Considering the influence of personal factors, health status emerged as an important parameter in the preparedness to use assistive technologies for health-related purposes. Healthy individuals reached higher means than chronically ill ones regarding the perceived advantages of such technical innovations in general, but this effect was also especially evident in the willingness to use age-appropriate functions (e.g., frailty monitoring). Gender showed its influence when it comes to disadvantages of technology: Male participants felt generally less restricted and were significantly more inclined to allow technology to assist them in their everyday life as opposed to the female respondents.

Overall, the acceptance of assistive technology in the life of older adults is particularly determined by the associated perceptions of pros and cons. As the general technology acceptance strongly correlates with the concrete applications, chances are that the today's elders are thoroughly prone to the use of health-supporting technologies; but the technologies have to be well-adjusted to their needs and enable their self-determined and independent living at home, preventing their social disconnectedness—an extremely valuable factor for the well-being and mental health.

### Integration of Knowledge About Patients' Concepts Into Medical Practice

Our results approve the geriatric core objectives of autonomy with large independence from caregivers (especially outside the family) as well as from institutionalized nursing care. For that, both the individual self-perception and professional geriatric assessment have to bring out the positive aspects of functioning prior to aspects of disability and deficits (61). That salutogenic emphasis might contribute to a rather “pro-aging” than “anti-aging” attitude in accordance to what we have observed in the first part of our study (see section Nursing Preferences). Receiving professional nursing care is perceived as interference in private life with rather negative connotations. It is a pending research question as to how professional (i.e., non-familial) caregivers can replace the needs on a interpersonal level to some degree cushioning singularization, loneliness, and social isolation (62). In Germany, still about a half of all dependent older adults receive care by the own family members (63). Already supported by different legislative measures throughout Europe in terms of a “family caregiver leave,” the obviously favored option of a trustful intrafamilial and intergenerational solidarity (see section Handling of Care in the Family) might be conceptually broadened by the resurgence of multigenerational houses (64). In this context, future research should investigate country-specific differences particularly focusing on the perceptions of aging and care as well as preferences related to the provided nursing care. These potential differences should be analyzed in light of the socio-cultural and policy-related circumstances of the respective countries as they might provide some insightful explanations of existing similarities and differences.

Finally, from a medical point of view our study shows that assistive technology is in general highly accepted to selectively optimize and compensate for activity and participation (see section General Acceptance). This also holds true for early hazard recognition and alert in community-dwelling settings with a general openness for digital measures as long as they do not require personal imaging (see section Acceptance of Specific Assistive Applications and Functions). Obviously, as long as camera-based techniques are directly associated with realistic depiction (which technically need not be the case), they will be perceived as an unacceptable infringement of privacy notwithstanding their advantages of comfortable unobtrusiveness and their developmental potential in terms of fall detection or vital sign monitoring as early warning against acute deterioration in health.

### Limitations and Future Research Directions

Eventually, some restrictions of this study and the need for future research should be pointed out. First, a higher case number of the oldest individuals (85+ years) is highly desirable. Even though, this study entails participants who reached this wonderful age, the total number of individuals in this age group is too low to be statistically compared to younger individuals in their late adulthood. This may engender a methodological artifact that let us overlook the existing differences between younger and older seniors, because the age groups were assigned by means of median rather than according to the geriatric classification of the older adults' age groups. Second, collection of higher case numbers of acutely ill persons would be very advantageous to enlarge knowledge upon to what extent health status and, if applicable, whether certain diseases have more influence on the acceptance and the use of assistive technologies. Here, longitudinal studies would provide an optimal future research framework. Another methodological shortcoming must be considered when interpreting the findings: Especially among the older individuals, a lack of access to, or ability to complete, an online survey by all persons concerned can falsify the identification of the real needs. This fact implies that the use of assistive technology primarily reaches an already selected group of older adults, who are familiar with technological innovations. To meet the needs of less technology-savvy persons in this target group, and thereby increase the representativeness, future research has thus to consider the traditional paper and pencil data collection in addition to the online survey method.

## Conclusion

Insights gained from this study enrich the existing knowledge about the perceptions of aging from the geriatric and the social perspective. A major strength of this study is that data were taken from an international survey presenting opinions of individuals (60+ years) from different cultures. Most older adults are open to technical innovations which support them to maintain their health and well-being, make their everyday life easier, and ensure their independence as far as possible. According to the findings, care preferences clearly contribute to a successful adoption of assistive technologies in this target group. Among the examined personal characteristics, health status, and gender significantly influence opinions regarding topics associated with aging. Consistent consideration of these findings can be excellently integrated into everyday clinical practice, whereby many procedures and processes can be facilitated and supported.

## Data Availability Statement

The raw data supporting the conclusions of this article will be made available by the authors on reasonable request and with the permission of the funding organization.

## Author Contributions

WW led the overall study, contributed to the data collection, analysis and interpretation, and wrote the most part of the manuscript. JO-vH wrote a part of the manuscript, contributed to the data collection, and manuscript edits. TL contributed to study design and manuscript edits. LB wrote a part of the manuscript and manuscript edits. MZ contributed to the data interpretation and manuscript edits. All authors read, contributed to the research design, and approved the final manuscript.

## Conflict of Interest

The authors declare that the research was conducted in the absence of any commercial or financial relationships that could be construed as a potential conflict of interest.
